# Amantadine Combines Astroglial System Xc^−^ Activation with Glutamate/NMDA Receptor Inhibition

**DOI:** 10.3390/biom9050191

**Published:** 2019-05-17

**Authors:** Tomosuke Nakano, Toshiki Hasegawa, Dai Suzuki, Eishi Motomura, Motohiro Okada

**Affiliations:** Department of Neuropsychiatry, Division of Neuroscience, Graduate School of Medicine, Mie University, Tsu 514-8507, Japan; t-nakano@clin.medic.mie-u.ac.jp (T.N.); t-hasegawa@clin.medic.mie-u.ac.jp (T.H.); dsky@clin.medic.mie-u.ac.jp (D.S.); motomura@clin.medic.mie-u.ac.jp (E.M.)

**Keywords:** amantadine, system xc^−^, L-glutamate, glutathione, astrocyte

## Abstract

A glutamate/NMDA receptor (NMDA-R) antagonist, amantadine (AMA) exhibits a broad spectrum of clinically important properties, including antiviral, antiparkinsonian, neuroprotective, neuro-reparative and cognitive-enhancing effects. However, both clinical and pre-clinical studies have demonstrated that noncompetitive NMDA-R antagonists induce severe schizophrenia-like cognitive deficits. Therefore, this study aims to clarify the clinical discrepancy between AMA and noncompetitive NMDA-R antagonists by comparing the effects of AMA with those of a noncompetitive NMDA-R antagonist, MK801, on rat tripartite glutamatergic synaptic transmission using microdialysis and primary cultured astrocytes. Microdialysis study demonstrated that the stimulatory effects of AMA on L-glutamate release differed from those of MK801 in the globus pallidus, entorhinal cortex and entopeduncular nucleus. The stimulatory effect of AMA on L-glutamate release was modulated by activation of cystine/glutamate antiporter (Sxc). Primary cultured astrocytes study demonstrated that AMA also enhanced glutathione synthesis via Sxc activation. Furthermore, carbon-monoxide induced damage of the astroglial glutathione synthesis system was repaired by AMA but not MK801. Additionally, glutamate/AMPA receptor (AMPA-R) antagonist, perampanel enhanced the protective effects of AMA. The findings of microdialysis and cultured astrocyte studies suggest that a combination of Sxc activation with inhibitions of ionotropic glutamate receptors contributes to neuroprotective, neuro-reparative and cognitive-enhancing activities that can mitigate several neuropsychiatric disorders.

## 1. Introduction

An approved glutamate/NMDA receptor (NMDA-R) antagonist, amantadine (AMA), exhibits a broad spectrum of clinically important properties, including antiviral and antiparkinsonian effects. It has been established that blocking the M2 small viral membrane protein is an element of antiviral activity of AMA [1], and inhibiting both NMDA-R and catecholamine transporter plays an important role in the antiparkinsonian effect of AMA [2,3]. In advanced Parkinson’s disease, AMA is utilized primarily to treat peak-dose L-DOPA-induced dyskinesia [4]. Notably, AMA was found to improve speech disturbance in advanced Parkinson’s disease patients with subthalamic nucleus deep brain stimulation resistance [4].

AMA is a commonly prescribed medication for its off-label uses such as the treatment of disorders of consciousness in patients who are undergoing inpatient neurorehabilitation [5]. AMA was also observed to accelerate the pace of functional recovery during active treatment in patients with post-traumatic disorders of consciousness [6]. Furthermore, AMA and memantine, which is a dimethyl derivative of AMA [7], have improved the cognitive impairments associated with several psychiatric disorders, including mood and anxiety disorders and schizophrenia [8,9,10]. This clinical evidence suggests that AMA not only leads to improvement in parkinsonism but also may exert cognitive-enhancing, neuroprotective and neuro-reparative effects.

The clinical effectiveness of AMA on several neuropsychiatric symptoms cannot be fully explained by its NMDA-R antagonism, since both clinical and pre-clinical studies have demonstrated that inhibition of NMDA-R induces severe cognitive dysfunctions [11,12,13]. Recently, we demonstrated that an NMDA-R antagonistic cognitive enhancer, memantine activates astroglial cystine/glutamate antiporter (Sxc) [7]. Clinical studies have reported that an Sxc activator N-acetyl-L-cysteine (NAC) partially improves cognitive dysfunction in several neuropsychiatric disorders [14,15]. Sxc is known to be a rate-limiting molecule in glutathione production [16], and it is capable of preventing neuronal damage induced by the RedOx response [16]. These effects of memantine—inhibition of NMDA-R and activation of Sxc—probably contribute to its clinical action of cognitive improvement with neuroprotective effects [7].

On the basis of the above clinical and pre-clinical findings, this study aims to clarify the novel mechanisms of clinical effect of AMA, including cognitive-enhancing, neuroprotective, neuro-reparative and antiparkinsonian effects. To this end, in the present study, the effects of AMA on tripartite glutamatergic transmission, including its effects on NMDA-R and Sxc, were determined using microdialysis and primary cultured astrocytes and compared with the effects of a noncompetitive and selective NMDA-R antagonist, MK801. The major results of this study indicate that AMA enhances the astroglial synthesis of the neuroprotective reduced form of glutathione via astroglial Sxc activation, similar to the observed effects of memantine [7]. Furthermore, the neuroprotective and neuro-reparative actions of AMA were demonstrated by examining the effects of AMA on an in vitro model of carbon monoxide (CO)-induced astrocyte damage. Activation of neuroprotective glutathione combined with inhibition of neurotoxic ionotropic glutamate receptors, including NMDA-R and glutamate/AMPA receptor (AMPA-R), protects the astroglial glutathione synthesis system from carbon monoxide poisoning (COP)-induced damages.

## 2. Materials and Methods

### 2.1. Chemical Agents

The NMDA-R antagonists, MK801 and amantadine (AMA) [17], and a cysteine prodrug, N-acetyl-L-cysteine (NAC) [18] were obtained from Wako Chemicals (Osaka, Japan). The cystine/glutamate antiporter (Sxc) inhibitor (S)-4-carboxyphenylglycine (CPG) [19] was purchased from Tocris Bioscience (Bristol, UK). Selective group I metabotropic glutamate receptors (I-mGluRs) antagonist, YM298198, was purchased from Sigma-Aldrich (St. Louis, MO, USA). A selective AMPA-R antagonist, perampanel (PER), was obtained from Cosmo Bio (Tokyo, Japan). All compounds were prepared on the day of their use in experiments. MK801, AMA, YM298198 and NAC were dissolved in modified Ringer’s solution (MRS) or artificial cerebrospinal fluid (ACSF). PER was initially dissolved at a concentration of 1 mM in dimethyl sulfoxide. CPG was initially dissolved in 1 N NaOH and was diluted to 1 μM in MRS.

### 2.2. Preparation of Microdialysis System

All animal care and experimental procedures described in this report complied with the Ethical Guidelines established by the Institutional Animal Care and Use Committee at Mie University (No.29-22). All studies involving animals are reported in accordance with the ARRIVE guidelines for reporting experiments involving animals [20]. A total of 102 rats were used in the experiments described here.

Male Sprague-Dawley rats (approximately 250 g, SLC, Shizuoka, Japan) were maintained in a controlled environment (22  ±  1 °C) on a 12-h dark/12-h light cycle. Rats were anesthetized with 1.8% isoflurane and then placed in a stereotaxic frame for 1 h. Concentric direct insertion type dialysis probes were implanted in the globus pallidus (in humans, simply known as the external globus pallidus) (A = −1.5 mm, L = 2.4 mm, V = −7.6 mm, relative to the bregma) (0.22 mm diameter, 2 mm exposed membrane: Eicom, Kyoto, Japan) [21,22], the entorhinal cortex (A = −8.0 mm, L = 4.8 mm, V = −7.0 mm, relative to the bregma) (0.22 mm diameter, 2 mm exposed membrane: Eicom, Kyoto, Japan) [23] and the entopeduncular nucleus (in humans, known as the internal globus pallidus) [24] (A = −2.4 mm, L = −2.8 mm, V = −8.0 mm, relative to bregma) (0.22 mm diameter, 1 mm exposed membrane: Eicom) [21,22] at a lateral angle of 25° [24]. Following surgery, rats were housed individually in cages during recovery and experiments, with food and water given ad libitum.

Perfusion experiments commenced 18 h after recovery from isoflurane anesthesia [25]. The rat was placed into the system for freely moving animals (Eicom) equipped with a two-channel swivel (TCS2-23; ALS, Tokyo, Japan). The perfusion rate was set at 1 μL/min in all experiments, using MRS [26,27]. MRS composed of (in mM) 145 Na^+^, 2.7 K^+^, 1.2 Ca^2+^, 1.0 Mg^2+^ and 154.4 Cl^−^, buffered to pH 7.4 with 2 mM phosphate buffer and 1.1 mM Tris buffer [26,27]. Dialysate was collected every 20 min. Extracellular L-glutamate level was measured at 8 h after starting the perfusion. The microdialysis experiments were carried out on awake and freely moving rats. To determine the effects of each agent, the perfusion medium was switched to MRS containing the target agent. Each dialysate was injected into the ultra-high-pressure liquid-chromatography (UHPLC) apparatus.

### 2.3. Preparation of Primary Astrocyte Culture

Astrocytes were prepared using a protocol adapted from previously described methods [7,28,29,30]. Our previous studies demonstrated that the remaining adherent cells included 95% GFAP-positive and A2B5-negative cells, as detected using immunohistochemical staining [7,28,29,30]. Cortical astrocyte cultures were prepared from neonatal Sprague-Dawley rats (SLC) (*n* = 24) sacrificed by decapitation at 0–24 h of age and removal cerebral hemispheres under dissecting microscope. Tissue was chopped into fine pieces using scissors and then triturated briefly with micropipette. Suspension was filtered using 70 µm nylon mesh (BD, Franklin Lakes, NJ, USA) and centrifuged. Pellets were re-suspended in 10 mL Dulbecco’s modified Eagle’s medium containing 10% fetal calf serum (fDMEM) (repeated three times). After 14 days culture (DIV14), contaminating cells were removed by shaking in standard incubator for 16 h at 200 rpm. On DIV21, astrocytes were removed from flasks by trypsinization and seeded onto translucent PET membrane (1.0 μm) with 24-well plates (BD) directly at a density of 105 cells/cm^2^ for experiments [7,28]. During DIV21~DIV28, the culture medium was changed twice a week. On DIV28, cultured astrocytes were washed out using ACSF (repeated three times) (wash-out).

To study effects of AMA on Sxc activity, after the wash-out, astrocytes were incubated in ACSF (100 μL) at 35 °C for 60 min in CO_2_ incubator (pre-treatment incubation). After pre-treatment incubation, astrocytes were incubated in ACSF containing AMA (0.3–100 μM) or cystine (0–400 μM) for 60 min and ACSF was collected for analysis of levels of L-glutamate and D-serine [7]. ACSF composed of (in mM) NaCl 130 mM, KCl 5.4 mM, CaCl_2_ 1.8 mM, MgCl_2_ 1 mM, and glucose 5.5 mM, and buffered with 20 mM HEPES buffer to pH 7.3 [28].

The effects of the interaction between glutamate receptor antagonists (AMA, MK801 and PER) and CO on astroglial glutathione synthesis was studied in incubating astrocytes according to the following four experimental designs. (1) Astrocytes were cultured in fDMEM containing AMA (0.3–100 μM) for 7 days (DIV21–28), (2) astrocytes were cultured in fDMEM containing AMA (10 μM), MK801 (1 μM), PER (1 μ), AMA (10 μM) + MK801 (1 μM) or AMA (10 μM) + PER (1 μM) for 7 days (DIV21~28) (non CO-exposure administration), (3) on DIV21, after astrocytes were incubated in 0.3% CO for 8 h according to previously published CO-exposure model [31], astrocytes were cultured in fDMEM containing AMA (10 μM), MK801 (1 μM), PER (1 μM), AMA (10 μM)+MK801 (1 μM) or AMA (10 μM) + PER (1 μM) for 7 days (DIV21–28) (post CO-exposure administration), (4) on DIV21, astrocytes were cultured in fDMEM containing AMA (10 μM), MK801 (1 μM), PER (1 μ), AMA (10 μM) + MK801 (1 μM) or AMA (10 μM) + PER (1 μM) for 3 h before 0.3% CO-exposure. After 8 h of 0.3% CO-exposure [31] in fDMEM containing the same agents, astrocytes were cultured fDMEM containing the same agents for 7 days (DIV21–28) (pre CO-exposure administration). On DIV28, after wash-out, astrocytes were lysed via sonicator [32]. Intra-astroglial glutathione level was determined using UHPLC with mass spectrometry (UHPLC/MS).

### 2.4. UHPLC and UHPLC/MS

Levels of L-glutamate and D-serine in MRS and ACSF were determined by UHPLC (xLC3185PU, Jasco, Tokyo, Japan) with fluorescence resonance energy transfer detection (xLC3120FP, Jasco) after dual derivatization with isobutyryl-L-cysteine and o-phthalaldehyde [7]. Derivative reagent solutions were prepared by dissolving isobutyryl-L-cysteine (2 mg) and o-phthalaldehyde (1 mg) in 0.1 mL ethanol followed by the addition of 0.9 mL sodium borate buffer (0.2 M, pH 9.0). Automated pre-column derivative was carried out by drawing up a 5 μL aliquot sample, standard or blank solution, and 5 μL of derivative reagent solution, and holding in reaction vials for 5 min before injection. The derivatized samples (5 μL) were injected by auto sampler (xLC3059AS, Jasco,). Analytical column (YMC Triat C18, particle 1.8 μm, 50 × 2.1 mm, YMC, Kyoto, Japan) was maintained at 45 °C and flow rate was set at 500 μL/min. A linear gradient elution program was performed over 10 min with mobile phase A (0.05 M citrate buffer, pH 5.0) and B (0.05 M citrate buffer containing 30% acetonitrile and 30% methanol, pH 3.5). The excitation/emission wavelengths of fluorescence detector were set at 345/455 nm [33,34].

For the determination of glutathione level, 5 μL aliquots of filtered samples were injected to the UHPLC/MS system (Acquity; Waters, Milford, MA, USA) with a Triat C18 column (particle 1.8 μm, 50 × 2.1 mm; YMC) that was maintained at 40 °C. The mobile phase was set at 500 μL/min and consisted of a 10 min linear gradient of mobile phases A (0.1% acetate) and B (acetonitrile). Nitrogen flows for desolvation and in the cone were set at 750 and 50 L/h, respectively, and the desolvation temperature was set at 450 °C. The cone voltage for determination of glutathione (*m*/*z* = 308.2) was 30 V.

### 2.5. Statistical Analysis

All experiments were designed equal sizes (*n* = 6) per groups. All values were expressed as mean ± SD. A *p* value less than 0.05 was considered statistically significant. The microdialysis study were compared by linear mixed effects model (LME) using SPSS for Windows (ver 25, IBM, Armonk, NY, USA) followed by Tukey’s post hoc test using BellCurve for Excel (Social Survey Research Information Co., Tokyo, Japan), when the F-value of LME was significant. To represent the statistical significance of drug factor compared with LME and Tukey’s post hoc test, the data (levels of L-glutamate) was expressed as the area under the curve (AUC_20–180 min_) values. Effects of AMA on L-cystine-induced astroglial releases of L-glutamate and D-serine were two-way analysis of variance with Tukey’s post hoc test (BellCurve for Excel). Concentration-dependent effects of AMA on astroglial Sxc activity and glutathione synthesis of primary cultured astrocytes were analyzed by logistic regression analysis (BellCurve for Excel). Interaction between glutamate receptor antagonists (AMA, MK801 and PER) and CO on intra-astroglial glutathione level was analyzed by two-way analysis of variance with Tukey’s post hoc test (BellCurve for Excel).

## 3. Results

### 3.1. Microdialysis Study

#### 3.1.1. Effects of Systemic Administration of MK801 and AMA on Extracellular L-glutamate Levels in the Globus Pallidus, Entorhinal Cortex and Entopeduncular Nucleus.

**Figure 1:**
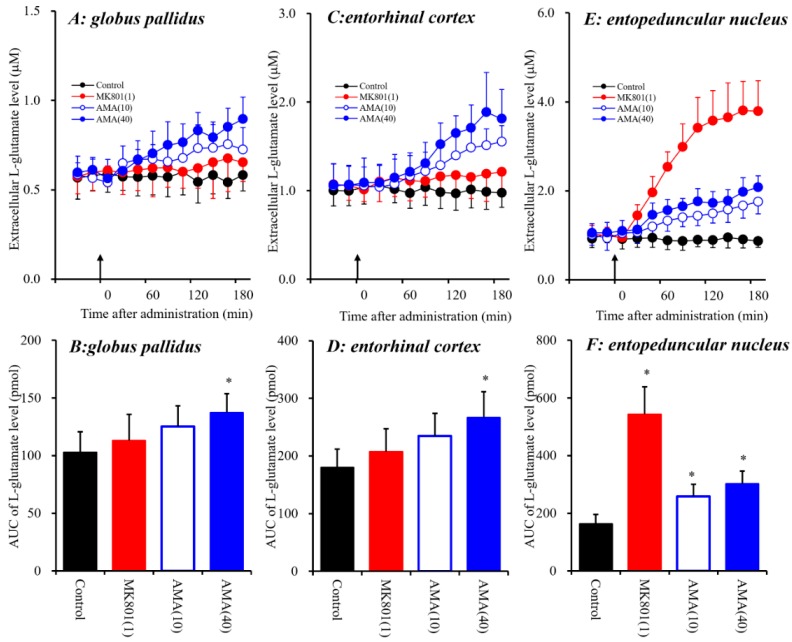
Effects of systemic administration of MK801 (1 mg/kg, i.p.) and amantadine (AMA) (10 and 40 mg/kg, i.p.) on extracellular L-glutamate levels in the globus pallidus (**A**,**B**), entorhinal cortex (**C**,**D**) and the entopeduncular nucleus (**E**,**F**). (**A**,**C**,**E**) indicate the effects of intraperitoneal administration of MK801 and AMA (arrows) on extracellular L-glutamate levels in the globus pallidus, entorhinal cortex and entopeduncular nucleus, respectively. The initial perfusion medium was modified Ringer’s solution (MRS). After the stabilization of L-glutamate levels in the perfusate, each rat was administrated with MK801 or AMA (i.p.). Ordinates: mean ± SD (*n* = 6) of extracellular L-glutamate level (μM); abscissa: time after administration of the target agent (min). (**B**,**D**,**F**) indicate the area under curve (AUC) value of extracellular L-glutamate levels (pmol) from 20 to 180 min after administration of the target agent in (**A**,**C**,**D**). * *p* < 0.05 relative to the control by a linear mixed model (LME) with Tukey’s post hoc test.

For the present study, three brain regions were selected for analysis, the globus pallidus, entorhinal cortex and entopeduncular nucleus. The entorhinal cortex plays important roles in attention, conditioning, event and spatial cognition, and it possibly exerts its actions by compressing representations that overlap in time [35]. The globus pallidus contacts with widespread cortical and basal ganglia regions, and contributes to various functions, including movement, motivation and cognition [36]. Especially, Lapresle and Fardeau identified COP-induced necrosis in the globus pallidus, demyelination in the cerebral white matter, and spondylotic changes in the cerebrum, including the entorhinal cortex [37]. Damage in the globus pallidus is frequently seen in patients with COP [38]. Therefore, in this study, the effects of AMA and MK801 on glutamatergic transmission in the globus pallidus, entorhinal cortex and entopeduncular nucleus were compared using microdialysis in freely moving rats (Figure 1) to determine the effects of intraperitoneal (i.p.) administrations of the NMDA-R antagonist MK801 (1 mg/kg, i.p.) [25,39] and therapeutic-relevant dose of AMA (10 and 40 mg/kg, i.p.) [40] on extracellular L-glutamate levels in the entorhinal cortex, globus pallidus and entopeduncular nucleus.

A dose of 40 mg/kg AMA was chosen because it has proven effective in the reducing axial, forelimb, and orolingual abnormal involuntary movements in 6-hydroxydopamine hemi-lesioned rats without affecting the locomotive components [40]. A dose of 1 mg/kg MK801 was chosen because it was shown to completely suppress orphenadrine-induced status convulsive epileptics [39]. The globus pallidus consists of two regions: the globus pallidus (in humans, simply known as the external globus pallidus) and the entopeduncular nucleus (in humans, known as the internal globus pallidus) [24].

Systemic administration of AMA (10 and 40 mg/kg, i.p.) affected extracellular L-glutamate levels in the globus pallidus [F_AMA_(2,15) = 5.0 (*p* < 0.05), F_Time_(9,135) = 30.8 (*p* < 0.01), F_AMA*Time_(18,135) = 13.8 (*p* < 0.01)], the entorhinal cortex [F_AMA_(2,15) = 6.4 (*p* < 0.01), F_Time_(9,135) = 72.9 (*p* < 0.01), F_AMA*Time_(18,135) = 25.5 (*p* < 0.01)], and the entopeduncular nucleus [F_AMA_(2,15) = 16.0 (*p* < 0.01), F_Time_(9,135) = 108.8 (*p* < 0.01), F_AMA*Time_ (18,135) = 35.5 (*p* < 0.01)] (Figure 1). Contrary to AMA, systemic administration of MK801 (1 mg/kg, i.p.) did not affect extracellular L-glutamate levels in the globus pallidus or entorhinal cortex, but increased the levels in the entopeduncular nucleus [F_MK801_(1,10) = 77.7 (*p* < 0.01), F_Time_(9,90) = 91.3 (*p* < 0.01), F_MK801*Time_(9,90) = 98.1 (*p* < 0.01)] (Figure 1E,F). The discrepancy between the effects of systemic administrations of AMA and MK801 (a selective NMDA-R antagonist) on L-glutamate releases in the globus pallidus, entorhinal cortex and entopeduncular nucleus suggests that the effects of a therapeutic-relevant dose of AMA on L-glutamate release in these three regions are mediated by mechanisms other than NMDA-R antagonism (see detailed mechanisms in discussion Section 4.1).

#### 3.1.2. Effects of Local Administration of NAC and CPG into the Globus Pallidus, Entorhinal Cortex and Entopeduncular Nucleus on Extracellular L-glutamate Levels.

**Figure 2:**
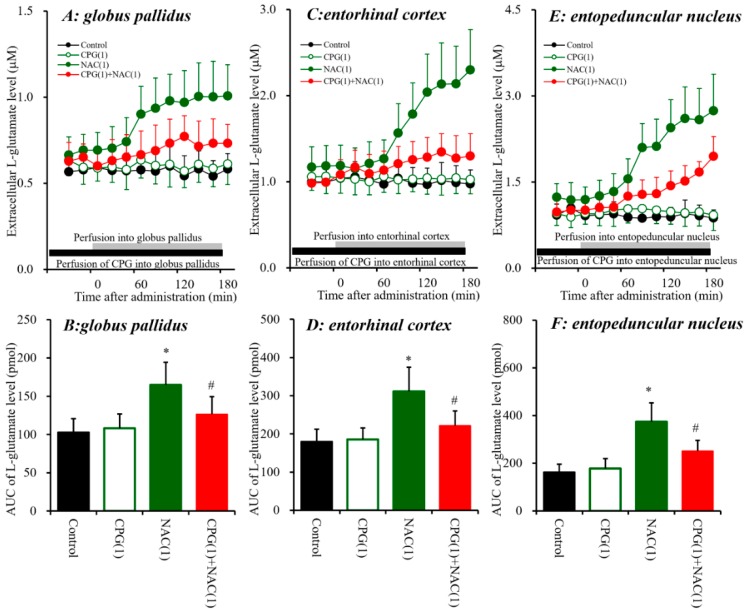
Effects of local administration of N-acetyl-L-cysteine (NAC) (1 mM) and (S)-4-carboxyphenylglycine (CPG) (1 μM) into the globus pallidus, entorhinal cortex and entopeduncular nucleus on the respective extracellular L-glutamate levels in the globus pallidus (**A**,**B**), entorhinal cortex (**C**,**D**), and entopeduncular nucleus (**E**,**F**). (**A**,**C**,**E**) indicate the effects of perfusion with NAC and CPG into the globus pallidus, entorhinal cortex and entopeduncular nucleus on extracellular L-glutamate levels, respectively. The initial perfusion medium was MRS. After the stabilization of L-glutamate levels in the perfusate, the perfusion medium was switched from MRS to MRS containing CPG (1 μM) or NAC (1 mM) (gray bars). To determine the effects of CPG on NAC-induced L-glutamate release, the initial perfusion medium was MRS containing GPG (1 μM) (black bars). After the stabilization of L-glutamate levels in the perfusate, the perfusate was switched to MRS containing CPG (1 μM) plus NAC (1 mM) (gray bars). Ordinates: mean ± SD (*n* = 6) of extracellular L-glutamate level (μM); abscissa: time after administration of the target agent (min). (**B**,**D**,**F**) indicate the AUC values of extracellular L-glutamate levels (pmol) after 20 to 180 min perfusion with the target agent in (**A**,**C**,**D**). * *p* < 0.05 relative to the control, and # *p* < 0.05 relative to NAC by LME with Tukey’s post hoc test.

Systemic administration of AMA increased extracellular L-glutamate levels in the three regions; however, systemic MK801 administration increased L-glutamate levels in the entopeduncular nucleus without affecting those in the globus pallidus or entorhinal cortex (Figure 1). These discrepancies between the effects of AMA and MK801 on L-glutamate release suggest that AMA pharmacologically affects regulation mechanisms other than NMDA-R systems; such potential mechanisms include those that involve Sxc, similar to the mechanisms of memantine, which is a dimethyl derivative of AMA [7]. Additionally, our previous study suggested that systemic MK801-induced L-glutamate releases in the frontal cortex were generated outside of the detected regions [7,25]. Therefore, to explore the stimulatory effects of systemic administration of AMA on extracellular L-glutamate levels in the globus pallidus, entorhinal cortex and entopeduncular nucleus, the effects of local administration of NAC (cysteine prodrug Sxc activator) [18] and (S)-4-carboxyphenylglycine (CPG: Sxc inhibitor) [41,42] into the globus pallidus, entorhinal cortex and entopeduncular nucleus were determined using microdialysis (Figure 2).

Similar to AMA, perfusion with NAC (1 mM) [7,25] into the globus pallidus, entorhinal cortex and entopeduncular nucleus increased extracellular L-glutamate levels in the globus pallidus [F_NAC_(1,10) = 13.4 (*p* < 0.01), F_Time_(9,90) = 31.4 (*p* < 0.01), F_NAC*Time_(9,90) = 34.0 (*p* < 0.01)], entorhinal cortex [F_NAC_(1,10) = 11.0 (*p* < 0.01), F_Time_(9,90) = 46.9 (*p* < 0.01), F_NAC*Time_(9,90) = 53.4 (*p* < 0.01)], and entopeduncular nucleus [F_NAC_(1,10) = 23.8 (*p* < 0.01), F_Time_(9,90) = 67.4 (*p* < 0.01), F_NAC*Time_(9,90) = 72.1 (*p* < 0.01)] (Figure 2). Contrary to NAC, perfusion with CPG (1 μM) [7,25] into the globus pallidus, entorhinal cortex, and entopeduncular nucleus did not affect the respective extracellular L-glutamate levels of these three regions (Figure 2), but inhibited NAC-induced L-glutamate release in the globus pallidus [F_CPG_(2,15) = 7.9 (*p* < 0.01), F_Time_(9,135) = 33.0 (*p* < 0.01), F_CPG*Time_(18,135) = 14.5 (*p* < 0.01)], entorhinal cortex [F_CPG_(2,15) = 10.7 (*p* < 0.01), F_Time_(9,135) = 71.2 (*p* < 0.01), F_CPG*Time_(18,135) = 41.8 (*p* < 0.01)], and entopeduncular nucleus [F_CPG_(2,15) = 16.5 (*p* < 0.01), F_Time_(9,135) = 116.7 (*p* < 0.01), F_CPG*Time_(18,135) = 44.8 (*p* < 0.01)] (Figure 2). Therefore, these data suggest that activation of Sxc in the globus pallidus, entorhinal cortex and entopeduncular nucleus increases L-glutamate release in these regions.

#### 3.1.3. Interaction between Local Administration of AMA and CPG into the Globus Pallidus, Entorhinal Cortex and Entopeduncular Nucleus on L-glutamate Release.

**Figure 3:**
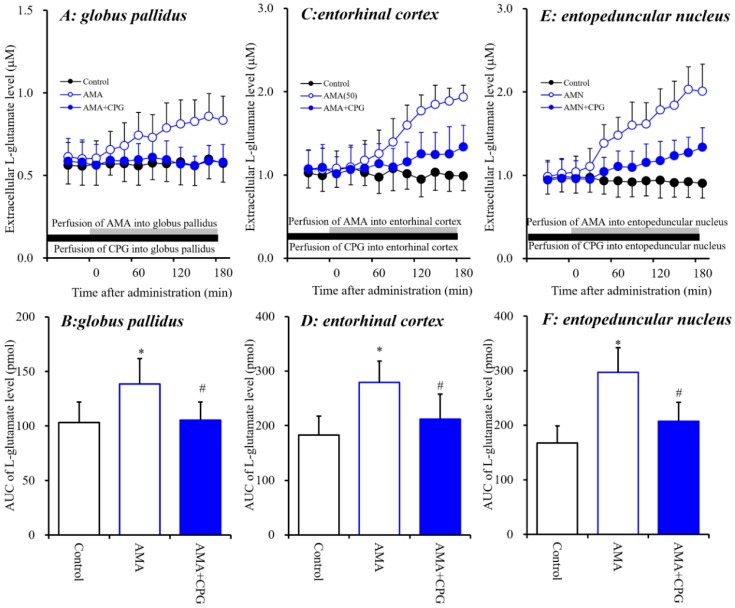
Interaction between perfusion with AMA (50 μM) and CPG (1 μM) into the globus pallidus, entorhinal cortex and entopeduncular nucleus and respective extracellular L-glutamate levels in the globus pallidus (**A**,**B**), entorhinal cortex (**C**,**D**) and entopeduncular nucleus (**E**,**F**). (**A**,**C**,**E**) indicate the effects of perfusion of AMA with or without CPG into the globus pallidus, entorhinal cortex and entopeduncular nucleus on extracellular L-glutamate levels, respectively. The initial perfusion medium was MRS with or without CPG (1 μM) (black bars). After the stabilization of L-glutamate levels in the perfusate, the perfusion medium was switched to the same MRS containing AMA (50 μM) (gray bars). Ordinates: mean ± SD (*n* = 6) of the extracellular L-glutamate level (μM); abscissa: time after administration of AMA (min). (**B**,**D**,**F**) indicate the AUC values of extracellular L-glutamate levels (pmol) after 20 to 180 min perfusion with the target agent in (**A**,**C**,**E**). * *p* < 0.05 relative to the control, and # *p* < 0.05 relative to AMA by LME with Tukey’s post hoc test.

The systemic administration of AMA and local administration of NAC increased extracellular L-glutamate levels in the three regions investigated (Figure 1 and Figure 2). Memantine exhibits weak inhibition of NMDA-R and activation of Sxc [7]. Therefore, to clarify the stimulatory effects of AMA on extracellular L-glutamate levels in the globus pallidus, entorhinal cortex and entopeduncular nucleus, the effect of interaction between local administration of AMA (50 μM) and Sxc inhibitor CPG (1 μM) on extracellular L-glutamate levels in the globus pallidus, entorhinal cortex, and entopeduncular nucleus was assessed (Figure 3).

A pharmacokinetic study in animal models demonstrated that the 50% effective AMA plasma concentration required to significantly reduce dyskinesia was around 10 μM across multiple species, from mice to nonhuman primates [10]. A recent clinical study reported that the plasma concentration of AMA in individuals administrated 161 mg/day AMA was around 3 μM [43]. In our previous study, the diffusion rates of various agents from perfusate to brain tissue ranged from 10% to 20% [7,25,44,45]. Taken together with the previous results, the estimated concentration of AMA ranged from 5 to 10 μM. Therefore, the perfusion concentration of 50 μM AMA is considered to be a therapeutic-relevant concentration.

The stimulatory effects of perfusion with AMA (50 μM) were inhibited by perfusion with CPG (1 μM) into the globus pallidus [F_CPG_(2,15) = 5.1 (*p* < 0.05), F_Time_(9,135) = 9.1 (*p* < 0.01), F_CPG*Time_(18,135) = 8.4 (*p* < 0.01)], entorhinal cortex [F_CPG_(2,15) = 7.5 (*p* < 0.01), F_Time_(9,135) = 68.5 (*p* < 0.01), F_CPG*Time_(18,135) = 42.9 (*p* < 0.01)] and entopeduncular nucleus [F_CPG_(2,15) = 15.6 (*p* < 0.01), F_Time_(9,135) = 106.5 (*p* < 0.01), F_CPG*Time_(18,135) = 54.1 (*p* < 0.01)] (Figure 3). These results strongly suggest that AMA increases L-glutamate release via activation of Sxc in these three regions.

#### 3.1.4. Interaction between Local Administration of AMA and YM298198 into the Globus Pallidus, Entorhinal Cortex and Entopeduncular Nucleus on L-glutamate Release.

**Figure 4:**
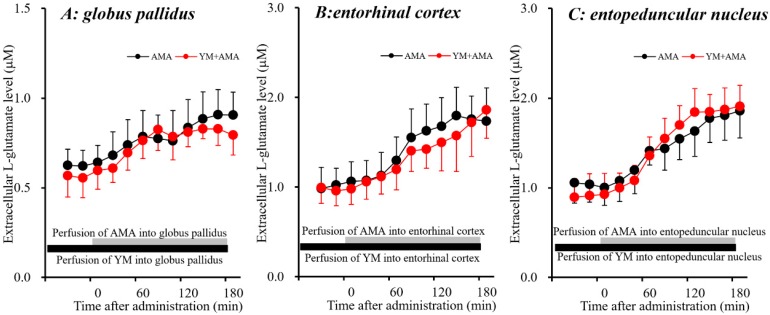
Interaction between perfusion with AMA (50 μM) and YM298198 (50 μM) into the globus pallidus, entorhinal cortex and entopeduncular nucleus and respective extracellular L-glutamate levels in the respective globus pallidus (**A**), entorhinal cortex (**B**) and entopeduncular nucleus (**C**). (**A**–**C**) indicate the effects of perfusion of AMA with or without YM298198 into the globus pallidus, entorhinal cortex and entopeduncular nucleus on extracellular L-glutamate levels. The initial perfusion medium was MRS with or without YM298198 (50 μM) (black bars). After the stabilization of L-glutamate levels in the perfusate, the perfusion medium was switched to the same MRS containing AMA (50 μM) (gray bars). Ordinates: mean ± SD (*n* = 6) of the extracellular L-glutamate level (μM); abscissa: time after administration of AMA (min). The significant effects of YM298198 on AMA-induced L-glutamate release was not detected by LME with Tukey’s post hoc test.

CPG is considered to be a useful Sxc inhibitor, whereas CPG also blocks group I metabotropic glutamate receptors (I-mGluRs). Therefore, the effects of a selective I-mGluRs inhibitor, YM298198 [46] on AMA-induced L-glutamate release in the globus pallidus, entorhinal cortex and entopeduncular nucleus were studied to clarify the mechanism of AMA-induced L-glutamate release. The stimulatory effects of perfusion with AMA (50 μM) were not affected by perfusion with YM298198 (50 μM) [46,47] in the globus pallidus, entorhinal cortex and entopeduncular nucleus (Figure 4). Therefore, pharmacologically AMA increases L-glutamate release in the globus pallidus, entorhinal cortex and entopeduncular nucleus via activation of Sxc but not I-mGluRs.

### 3.2. Primary Cultured Astrocyte Study

#### 3.2.1. Acute Effects of AMA on Astroglial Sxc Activity.

The stimulatory effects of AMA on extracellular L-glutamate levels in the globus pallidus, entorhinal cortex and entopeduncular nucleus were inhibited by perfusion with the Sxc inhibitor CPG, but not affected by I-mGluR inhibitor YM298198. The extracellular L-glutamate levels in the globus pallidus, entorhinal cortex and entopeduncular nucleus were increased by a cysteine prodrug, NAC. Pharmacologically, these results suggest that the AMA-induced release of L-glutamate is probably modulated by activation of astroglial Sxc. Our hypothesis was clarified by determining the extracellular concentration-dependent effects of L-cystine on L-glutamate release from rat primary cultured astrocytes.

**Figure 5:**
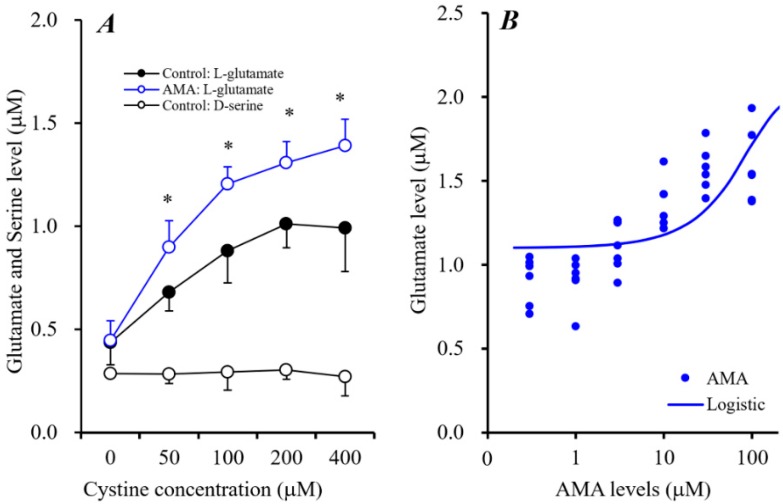
Concentration-dependent effects of L-cystine (0~400 μM) on astroglial release of L-glutamate and D-serine and effects of AMA (10 μM) on L-cystine-dependent L-glutamate release (**A**). Ordinate indicates mean ± SD (*n* = 6) of extracellular levels of L-glutamate or D-serine (μM); abscissa: concentration of L-cystine. * *p* < 0.05 relative to the control by two-way analysis of variance with Tukey’s post hoc test. Concentration-dependent effects of AMA (0–100 μM) on 100 μM L-cystine-induced astroglial L-glutamate release (**B**). Ordinate indicates mean ± SD (*n* = 6) of extracellular levels of L-glutamate (μM); abscissa: concentration of AMA (logistic regression analysis).

L-Cystine concentration-dependently increased astroglial L-glutamate release without affecting the release of D-serine (Figure 5A). L-Cystine-dependent release of astroglial L-glutamate was enhanced by 10 μM AMA [F_AMA_(1,5) = 64.6 (*p* < 0.01), Fc_ystine_(4,50) = 85.5 (*p* < 0.01), F_AMA*cystine_(4,50) = 4.5 (*p* < 0.01)] (Figure 5A). Moreover, Sxc activity (100 μM L-cystine-induced astroglial L-glutamate release) was concentration-dependently enhanced by AMA (0.3~100 μM) [F(1,35) = 25.0 (*p* < 0.01)] (Figure 5B). Therefore, AMA weakly enhances astroglial Sxc activity.

#### 3.2.2. Effects of AMA Administration for 7 Days on Intra-Astroglial Glutathione Level.

**Figure 6:**
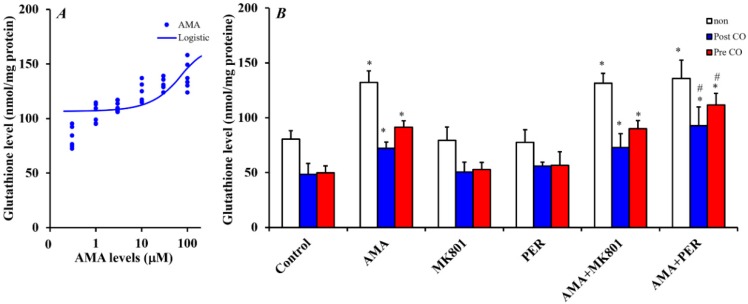
(**A**) Concentration-dependent effects of AMA administration (0~100 μM) for 7 days on the intra-astroglial glutathione level. Ordinate; mean ± SD (*n* = 6) of intra-astroglial glutathione level (nmol/mg protein); abscissa: concentration of AMA (μM) (logistic regression analysis). (**B**) Effects of AMA (10 μM), MK801 (1 μM) and PER (1 μM) on the intra-astroglial glutathione level in physiological conditions (non carbon monoxide (CO)), after CO-exposure administration (post CO) and before CO-exposure administration (pre CO). Ordinate; mean ± SD (*n* = 6) of intra-astroglial glutathione level (nmol/mg protein). * *p* < 0.05 relative to the control by two-way analysis of variance with Tukey’s post hoc test; # *p* < 0.05 relative to the AMA administrations by two-way analysis of variance with Tukey’s post hoc test [F_agent_(5,90) = 94.4 (*p* < 0.01), F_CO_(2,90) =151.4 (*p* < 0.01), F_agent*CO_(10,90) = 4.0 (*p* < 0.01)].

Sxc activity is a rate-limiting in the glutathione synthesis [16]. The effects of Sxc activation by AMA on glutathione production was explored by determining the concentration-dependent effects of AMA administration for 7 days on the intra-astroglial glutathione level. AMA (0.3~100 μM) increased the intra-astroglial glutathione level in a concentration-dependent manner [F(1,35) = 17.7 (*p* < 0.01)] (Figure 6A), whereas neither MK801 (1 μM) nor an AMPA-R antagonist, PER (1 μM) affected the intra-astroglial glutathione level (Figure 6B).

It has been demonstrated that CO-exposure inhibits glutathione synthesis [32]. Taken together with previous reports, the above results in this study suggest that the stimulatory effects of AMA on glutathione synthesis possibly contribute to its neuroprotective or neuro-reparative effects and repair of astroglial glutathione synthesis damage induced by CO-exposure. Astrocytes were cultured for 8 h according to previously published CO-exposure model (0.3%) [31]. After 7 days CO-exposure, the intra-astroglial glutathione level had decreased (60%) (Figure 6B). After the CO-exposure, the effects of AMA on CO-induced damage to the glutathione synthesis system were clarified by administrating AMA (10 μM), MK801 (1 μM) and PER (1 μM) to astrocytes for 7 days (post CO-exposure administration). After post CO-exposure administration, AMA weakly increased intra-astroglial glutathione level, whereas neither MK801 nor PER affected the intra-astroglial glutathione level (Figure 6B).

From 3 h before to 7 days after CO-exposure (pre CO-exposure administration), administration of AMA (10 μM) increased the intra-astroglial glutathione level, but neither MK801 (1 μM) nor PER (1 μM) had this effect (Figure 6B). Interestingly, PER alone did not affect the intra-astroglial glutathione level in physiological (non CO-exposure) or pathological (pre and post CO-exposure administrations) conditions; however, PER increased the intra-astroglial glutathione level in pre and post CO-exposure administrations when PER administration was combined with AMA (Figure 6B).

## 4. Discussion

### 4.1. Effects of AMA and MK801 on L-glutamate Release in the Globus Pallidus, Entorhinal Cortex and Entopeduncular Nucleus

Systemic administration of MK801 increased L-glutamate release in the frontal cortex [7,25], however, local administration of MK801 directly into the frontal cortex did not affect regional L-glutamate release [7,25,33,48]. The discrepancy between the effects of systemic and local MK801 administration suggest that the target region responsible for MK801-induced frontal hyper-glutamatergic transmission lies outside the frontal cortex. The frontal cortex receives two major glutamatergic projections: one from other cortical regions and one from the mediodorsal thalamic nucleus [33,48,49]. Recently, we demonstrated that aripiprazole, memantine, and guanfacine, all of which improve cognitive function, normalized thalamocortical glutamatergic abnormalities in the medial prefrontal cortex and orbitofrontal cortex [7,25,34].

**Figure 7:**
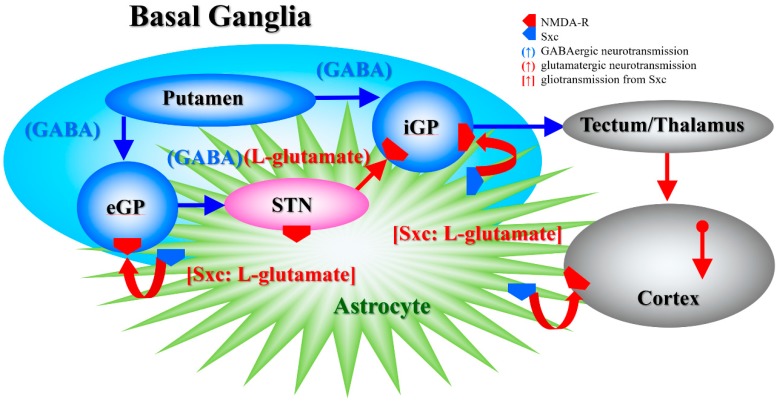
Our proposed hypothesis for the extended neural circuitry involved in the activation of L-glutamate release induced by MK801 and AMA in the internal globus pallidus (iGP: in rodents, the entopeduncular nucleus), external globus pallidus (eGP: in rodents, the globus pallidus) and entorhinal cortex. The putamen projects GABAergic terminals to both the eGP and iGP [50]. The eGP projects GABAergic terminals to the subthalamic nucleus (STN). The iGP receives glutamatergic projections from the STN and GABAergic projections from the putamen [50]. Cortical regions receive two major glutamatergic projections from other cortical regions and outside of the cortex. Inhibition of NMDA-R in the cortex did not affect L-glutamate release in previous studies [22,33,48,51], similar to the present results.

In the present study, systemic administration of therapeutic-relevant dose of AMA increased extracellular L-glutamate levels in the entopeduncular nucleus (known in humans as the internal globus pallidus), globus pallidus (known in humans as the external globus pallidus) and entorhinal cortex; however, systemic administration of MK801 drastically increased L-glutamate release in the entopeduncular nucleus without affecting that in the globus pallidus or entorhinal cortex. The lack of MK801-induced change in the extracellular levels of L-glutamate in the globus pallidus is easily understood, since the globus pallidus receives mainly inhibitory GABAergic terminals but few glutamatergic terminals from the putamen, and it projects GABAergic terminals to the subthalamic nucleus (Figure 7) [22,50]. Contrary to the globus pallidus, the entopeduncular nucleus receives both inhibitory GABAergic projections from the putamen and excitatory glutamatergic projections from the subthalamic nucleus which receives GABAergic inhibition from the globus pallidus (Figure 7) [22,50]. Therefore, the MK801-induced L-glutamate release in the entopeduncular nucleus is generated by activation of L-glutamate release from subthalamic nucleus projection which is induced by GABAergic disinhibition in the globus pallidus NMDA-R inhibition (Figure 7). The entorhinal cortex receives glutamatergic projections from other cortical regions (Figure 7) [23]. In particular, inhibition of cortical NMDA-R did not affect L-glutamate release in previous studies [48,51], similar to the present results. The detected discrepancy between the effects of systemic administrations of AMA and MK801 on the L-glutamate release in the globus pallidus and entorhinal cortex indicate, at least partially, that the AMA-induced regional elevation of L-glutamate release is probably not only generated by NMDA-R antagonism or neural circuits associated L-glutamate exocytosis mechanisms. To clarify the mechanisms of AMA-induced L-glutamate release, on the basis of our previous study [7], we determined the effects of AMA on Sxc associated L-glutamate release in this study.

### 4.2. Effects of AMA on Sxc Associated Transmission

In our recent study, memantine (dimethyl derivative of AMA) was determined to weakly activate astroglial Sxc activity [7]. On this basis, in the present study, the effects of AMA on extracellular L-glutamate associated with Sxc were determined using microdialysis. Activation of Sxc in the globus pallidus, entorhinal cortex and entopeduncular nucleus induced by local perfusion with the Sxc activator NAC increased extracellular L-glutamate levels in these three regions. NAC-induced L-glutamate release was inhibited by the Sxc inhibitor CPG. Similar to the effects of NAC, local perfusion with AMA into the globus pallidus, entorhinal cortex and entopeduncular nucleus also increased extracellular L-glutamate level, which was also inhibited by CPG but not by the selective I-mGluRs inhibitor YM298198. These results strongly suggest that AMA activates Sxc-associated L-glutamate release. Indeed, an in vitro study using primary cultured astrocytes demonstrated AMA effects that are similar to those of memantine [7]—that is, AMA enhanced the astroglial L-cystine-induced L-glutamate release.

It has been well established that CPG and NAC decrease and enhance, respectively, the synthesis of the neuroprotective antioxidant glutathione reduced form [16]. Glutathione is synthesized by glutamate/cysteine ligase and glutathione synthase from imported cystine, which is transported through the rate-limiting Sxc [16]. In this study, long-term (7 days) AMA administration increased the intra-astroglial glutathione level, which is probably the result of activated glutathione synthesis due to activation of Sxc, in both physiological and pathological (CO-exposure) conditions. These findings suggest the existence of multiple pharmacological targets of AMA, inhibition of NMDA-R, and enhancement of glutathione productions via activation of Sxc.

### 4.3. Effects of AMA on CO-Induced Astroglial Damage

CO is well known as a toxic molecule, because CO binds with high affinity to hemoglobin, resulting in hypoxia [52]. It has been well established that CO-induced hypoxia is a potentially a major mechanism of COP-induced neuronal damage in the acute phase [52,53]. The pathomechanism of COP-induced delayed neuropsychiatric sequelae is probably more complex than that of CO-induced hypoxia, since the former presents as recurrent severe neuropsychiatric symptoms that recur after an interval of apparent normality following the apparent resolution of acute symptoms [54]. COP-induced delayed neuropsychiatric sequelae directly affect the prognosis of survival for COP patients [54]. Unfortunately, there is no known medication to improve the prognosis of progression in severe COP-induced delayed neuropsychiatric sequelae [55]. Thus, the mainstay of medication for delayed neuropsychiatric sequelae is supportive care.

Recent pre-clinical studies have suggested that CO is a gaseous transmitter that resembles nitric oxide [53]. In particular, under physiological and pathological conditions, several pre-clinical studies have indicated that CO acts as a modulator of several ion channels, including voltage-sensitive sodium and calcium channels, hemichannels, and ionotropic glutamate receptors [56,57,58,59,60]. These CO-activated ion channels should enhance RedOx responses. CO induces apoptosis of neurons and astrocytes that contribute to COP-induced delayed neuropsychiatric sequelae or brain damage [61]. In spite of these observations, clinically, the free radical scavenger, edaravone administered alone could not improve any COP-induced chronic neuropsychiatric symptoms [62]. Glutathione is capable of preventing cell damage induced by the RedOx response [16], and glutathione protects against axonal demyelination (one of the major neuronal damage induced by COP) and enhances remyelination [63]. Therefore, the stimulatory effects of AMA on glutathione production probably repair/improve demyelination which is the one of the major COP-induced neurological injuries.

There are critical reports suggesting that activation of Sxc modulates the neuronal damage that occurs in several neuropsychiatric disorders. In particular, oxidative stress and hypoxic conditions have been shown to increase L-glutamate release and neuronal cell damage via activation of astroglial Sxc [16,64]. Inhibition of Sxc attenuated the cortical demyelination response in an autoimmune encephalomyelitis model through the reduction of Sxc-induced L-glutamate release [65]. Cystine imported through Sxc possibly contributes to a neuroprotective shift via glutathione synthesis, whereas counter-transported L-glutamate shifts toward neurotoxicity via activation of ionotropic glutamate receptors [64]. Glutathione inhibits the activities of both ionotropic glutamate receptors, NMDA-R and AMPA-R, via binding to their glutamate recognition sites [66,67]. In spite of the double-edge sword actions of Sxc/glutathione, AMA activates Sxc activity, but simultaneously prevents Sxc-induced neurotoxicity by NMDA-R inhibition. Indeed, in this study, AMA chronically increased the intra-astroglial glutathione level (likely the result of activated glutathione synthesis via activation of Sxc) in both physiological and pathological (CO-exposure) conditions. Contrary to AMA, neither MK801 nor PER affected astroglial glutathione production under physiological and pathological conditions, whereas PER enhanced the stimulatory effects of AMA on the glutathione level. These results support our hypothesis and suggest that the wide-range suppression of the RedOx response by enhancing glutathione is more effective than the selective suppression of ionotropic glutamate receptor in counteracting CO/COP-induced brain damage and delayed neuropsychiatric sequelae.

### 4.4. Clinical Implications of AMA Administration

Clinical studies have reported that the Sxc activator NAC partially improves cognitive dysfunction in several neuropsychiatric disorders [14,15]. Indeed, AMA has improved cognitive impairments in schizophrenia [8] and speech, gait or balance impairment in subthalamic nucleus deep brain stimulation-resistant advanced Parkinson’s disease patients [4]. Cognitive science has demonstrated that both the entorhinal cortex and globus pallidus play important roles in several types of neurocognitions [35,36]. The results of the present study cannot explain the detailed cognitive-enhancing mechanisms of AMA, but mild activation of extrasynaptic glutamatergic transmission via Sxc activation in both cognition generator regions, the entorhinal cortex and globus pallidus, probably contributes to its cognitive-enhancing effects. Notably, the pathomechanism of subthalamic nucleus deep brain stimulation resistance is considered to be associated with a disturbance of the subthalamic nucleus. The present results regarding AMA-induced L-glutamate release in the entopeduncular nucleus provide us with mechanisms that explain the clinical beneficial effects of AMA in the treatment of subthalamic nucleus deep brain stimulation resistance.

## 5. Conclusions

The present study, including in vivo microdialysis and in vitro primary cultured astrocyte studies, indicates a novel pharmacological target of AMA, weak enhancement of glutathione synthesis via activation of astroglial Sxc activity under the both physiological and pathological (CO-exposure) conditions. Contrary to stimulation of neuroprotective glutathione production, activation of Sxc probably increases the counter-transported neurotoxic L-glutamate release; however, its NMDA-R antagonism possibly prevents the neurotoxic response induced by Sxc. A combination of inhibition of NMDA-R and activation of Sxc, at least partially, contributes to the mechanisms of clinical action of AMA regarding neuroprotective and neuro-repair action of AMA.
